# Psychometric properties of the WHOQOL-BREF among next of kin to older persons in nursing homes

**DOI:** 10.1186/s12955-020-01345-9

**Published:** 2020-04-19

**Authors:** Helena Rosén, Gerd Ahlström, Annika Lexén

**Affiliations:** Department of Health Sciences, Faculty of Medicine, Lund University, PO Box 157, SE-221 00 Lund, Sweden

**Keywords:** Quality of life, Factor analysis, Confirmatory factor analysis

## Abstract

**Background:**

The worries of next of kin about their older loved ones in nursing homes can be extensive and can adversely affect their subjective experiences of their own physical, mental and social well-being. It is thus of utmost importance to measure the quality of life of next of kin in a valid and reliable way.

**Methods:**

The design is a cross-sectional study with psychometric evaluation based on classical test theory in preparation for a planned educational intervention study on palliative care. An abbreviated version of the World Health Organization’s quality-of-life self-assessment instrument WHOQOL, the Swedish WHOQOL-BREF, was completed by 254 next of kin of older persons in 30 nursing homes. Data quality was assessed via the mean, median, item response, missing values, and floor and ceiling effects. Reliability was estimated using Cronbach’s alpha and corrected item-total correlations. Construct validity was estimated by Spearman’s rank correlation, and model fit was assessed using confirmatory factor analysis.

**Results:**

The rate of missing data was low (less than 2%). Ceiling effects ranged from 11 to 43% and were above 20% for 21 of 24 items. The corrected item-total correlations varied between 0.35 and 0.68 and were thus well above the lower limit of 0.30. Cronbach’s alpha was 0.83, indicating satisfactory internal consistency. The confirmatory factor analysis indicated a fair to close model fit (comparative fit index 0.93, root mean squared error of approximation 0.06).

**Conclusions:**

The findings suggest that the WHOQOL-BREF may constitute a reliable and valid measure of quality of life for use among next of kin to older persons in nursing homes. When interpreting the results, it is important to assess the ceiling effect, as it may restrict the ability of the WHOQOL-BREF to detect true positive changes in quality of life over time.

**Trial registration:**

NCT02708498.

## Background

In countries with an ageing population, it is a common situation for an older person to live in a nursing home due to multiple morbidities with complex medical and care needs [1]. These frail older people need round-the-clock care for the remainder of their lives. Feelings of uncertainty and frustration among next of kin at admission is often related to experiences prior to the older person moving into the nursing home, and the initial period in the nursing home affects transition experiences for the next of kin [2]. The transfer to a nursing home means handing over a loved one into the care of the staff after a long period of providing care at home. Most next of kin remain actively involved in caregiving but are stressed by uncertainties about how to interact with the nursing home staff [3–6]. As ageing progresses, with increasingly severe multiple morbidities, this interaction can become even more stressful for the next of kin which may have a negative impact on their quality of life (QOL) [7, 8]. It may cause them to experience conflicting feelings of responsibility [9] between their own needs and those of their relative, as a result of taking part in the care while at the same time leaving the responsibility for care to the staff [9, 10] They often carry a heavy burden and may experience mental ill health [11]. They closely follow their loved one’s transition until the end of life, which has an impact on their own health and leads to difficulties in managing their daily lives [12]. Nursing homes have become a major arena for the provision of palliative care and little is known about the QOL of next of kin’s to older persons in nursing homes. Therefore, it is of utmost importance to measure their QOL in a reliable and valid way.

The WHO definition of QOL, applicable in this study, is a broad, multidimensional concept defined as individuals’ perceptions of their position in life in the context of the culture and value systems in which they live and in relation to their personal goals, expectations, standards and concerns [13–15] (WHOQOL Group, 1995, p. 1405). The experience of QOL varies over time and in different life situations. In conjunction with the WHO promotion of the *Health for All* goal, which includes mental, social and physical well-being in diverse populations around the globe, the organization began constructing a QOL assessment instrument in early 1990 [13]. The first published instrument, the WHOQOL-100, was developed for cross-cultural application and intended for international use. The instrument is based on 1) the WHO definition of QOL, 2) empirical evidence (such as focus groups with healthcare professionals, patients and healthy people) gathered by 15 international research centres representing different cultures and 3) statistical testing (such as test–retest reliability and structural equation modelling) demonstrating a four-domain structure [13, 16, 17]. Although the WHOQOL-100 provides a detailed assessment of individual QOL, it may be too lengthy for many respondents and less useful in a project where QOL is only one variable of interest [16, 17]. The WHO group therefore constructed the WHOQOL-BREF, based on the most general questions from each of the domains of the WHOQOL-100 [17]. The four WHOQOL-BREF domain scores correlated highly (0.89 or more) with the original WHOQOL-100 domain scores and had good discriminant and content validity, internal consistency and test–retest reliability [17]. Thus, it was concluded that the WHOQOL-BREF provides a valid and reliable alternative assessment to the WHOQOL-100, with good discriminant validity of the domain profiles [17].

A review of scientific publications shows that the WHOQOL-BREF is widely used. This instrument has been psychometrically tested on adult patients with a broad range of diseases and health conditions, from a variety of inpatient and outpatient somatic and psychiatric healthcare facilities, and on healthy people from the general population [18–26]. Examples of studies where the WHOQOL-BREF has been used are on wounded, injured and ill patients from the military [24], persons with Parkinson disease [21], and those with HIV or AIDS [26]. We have found only few published studies in the literature using WHOQOL-BREF to measure the QOL of next of kin. Two studies used QOL as an outcome measure of an intervention, one with mindfulness training for 130 next of kin of palliative inpatients in Germany [23], and one on using a telephone-based support program for 55 next of kin of patients with dementia in USA (20). Three studies investigated predictors of QOL among next of kin in different contexts: patients with a disorder of consciousness in Italy [19], patients with psychiatric illnesses in Jordan [18] and older persons aged 80 years and above in Brazil [27]. However, these studies are not psychometric evaluations and no study has been conducted specifically on next of kin to older persons living in nursing homes. The only studies measuring the psychometric properties of WHOQOL-BREF have been performed on patients [25, 28–30] and on the general population [25, 31]. Furthermore, the Swedish version of WHOQOL-BREF has not previously been tested on next of kin to older persons in nursing homes, although it has been used as an outcome measure in an educational intervention in palliative care in the Swedish KUPA (knowledge-based palliative care) project [32]. When performing intervention studies it is vitally important to have valid and reliable instruments that are sensitive enough to measure changes and to ensure that the established dimensionality and factor-loading pattern in WHOQOL-BREF fit the population in question in order to get reliable results. Accordingly, the purpose of this study was to investigate the psychometric properties of the WHOQOL-BREF among next of kin to older persons in nursing homes.

## Methods

This study is designed as a cross-sectional study with psychometric evaluation based on classical test theory [32].

### Research setting of the KUPA project

This study is part of a larger project of implementing palliative care in nursing homes, the KUPA project. In this project, an educational intervention for staff and managers is structured around the WHO definition of palliative care, which has been operationalized into four cornerstones: symptom relief for the patient, multi-professional cooperation, continuous communication and support to the patient and the family. The goal of palliative care in the KUPA project is thus to address physical, psychosocial and spiritual needs as well as to provide support to the family [33, 34]. The WHOQOL-BREF was used as the outcome measure for evaluating the impact of the intervention on the next of kin’s QOL. This instrument was chosen because it has a clear theoretical basis for the concept of QOL [17]. The project involved 30 nursing homes for older persons in the Swedish counties of Kronoberg and Skåne [32]. They were situated in both urban and rural areas and included a mix of large nursing homes (with more than 100 residents) and smaller ones (with fewer than 25 residents). The project and the current study were approved by the regional ethical review board in Lund, Sweden (approval no.: 2015/4), with the trial registration number NCT02708498**.**

### WHOQOL-BREF

The WHOQOL-BREF [17] consists of 24 items scored in four domains: physical health (7 items), psychological health (6 items), social relationships (3 items) and environment (8 items). The questionnaire also includes two items which are analysed separately: one question about overall assessment of QOL, and one about overall satisfaction with health. Each item has a 5-point response scale, and a higher score indicates better QOL. The domain score is calculated from the average score across the items in that domain.

The principal investigator of the KUPA project (second author GA) received permission to use the Swedish version of the WHOQOL-BREF by the WHO. The ethical review led to exclusion of one item, “*How satisfied are you with your sex life?*”, because it was deemed to be an unethical intrusion into the participant’s private life. This exclusion was also approved by the WHO.

#### Procedure

A contact person, who could be an assistant nurse, manager or administrator, at each nursing home was informed by a researcher about the study and its inclusion criteria and asked to make the initial contact with the next of kin. Those who fulfilled the inclusion criteria were informed about the study and then asked if they were interested in participating. Where this was the case, the contact person passed on the informal caregiver’s name and telephone number to the researcher, who then called each one and provided further information about the project and this particular study before inviting them to participate. Those who agreed to participate received the coded questionnaires by post with written information about the study and instructions on how to fill out the questionnaire. The package also included a consent form to sign and two prepaid envelopes, so that the consent form and the questionnaire could be returned separately to the researcher.

### Participants

**Fig. 1:**
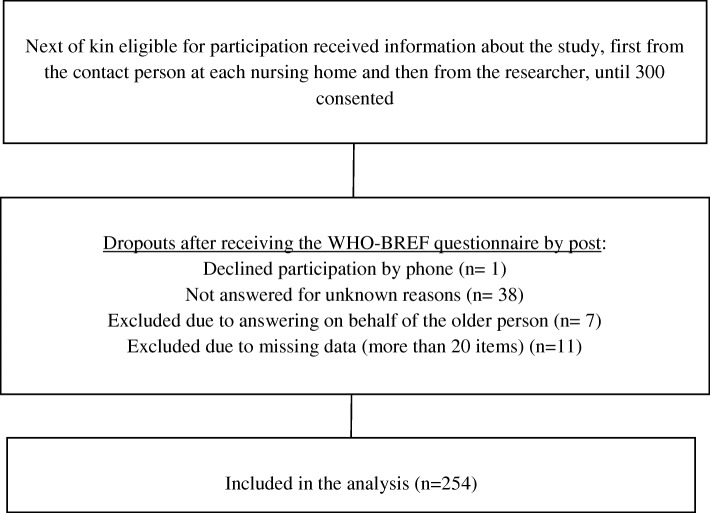
Flow chart showing the inclusion procedure for the study participants

The inclusion criterion for next of kin was having a relation to one of the residents, i.e., older persons living at the included nursing homes, but not necessarily being a family member. Additional inclusion criteria were that they had to be able to speak and understand Swedish, and did regularly visit the older person in the nursing home. The goal of recruitment was 5–10 participants per item, which means a total of 254 participants for this psychometric evaluation. Further individuals matching the inclusion criteria were invited to participate until 300 of them had given oral consent. This number was calculated on an expected dropout rate of 50 persons or 17%. The selected number of participants per nursing home was related to the capacity of the institution, ranging from 18 participants at a large nursing home to five participants at a small one. The number of dropouts and the reasons for dropout are shown in Fig. 1. Thus, the final sample consisted of 254 participants with a mean age of 64 years (SD = 9.7), mostly women (*n* = 191). Most participants (75%) usually visited the older person at the nursing home once a week or more. For more information on socio-demographic characteristics, see Table 1.

**Table 1:** Socio-demographic and clinical characteristics of 254 study participants (next of kin to older persons in nursing homes)

Age, mean (SD)	64 (9.7)
Gender, n (%)	
Men	63 (25)
Women	191 (75)
Civil status, n (%)	
Married/cohabiting	205 (81)
Divorced/single/living alone	42 (17)
Widow/widower	6 (2)
Relation, n (%)	
Wife	38 (15)
Child	192 (77)
Sibling	4 (2)
Grandchild	1 (1)
Friend	1 (1)
Other	10 (4)
Highest educational level, n (%)	
Compulsory school	51 (20)
Upper secondary school	62 (25)
Vocational qualification	41 (16)
University degree or equivalent	97 (39)
Employed, n (%)	
No	104 (41)
Yes, part time	55 (22)
Yes, full time	36 (37)
Visits to nursing home, n (%)	
A couple of times per year	2 (1)
Once a month or more	58 (23)
Once a week or more	167 (67)
Every day	21 (9)

### Data analysis

Data analysis was conducted using IBM SPSS Statistics v.20 and IBM SPSS Amos v.25 (IBM Corp., Armonk, NY, USA). In a first step, negatively phrased items in WHOQOL-BREF (Q3, Q4 and Q25) were reversed (1 = 5, 2 = 4, 3 = 3, 4 = 2, 5 = 1). Two items – one question about overall QOL and one about overall health – were analysed separately.

#### Validity

Internal validity in terms of data quality and targeting was assessed by missing values, item response, and floor and ceiling effects. Floor and ceiling effects were determined from the proportion of responses at the minimum and maximum extremes of the scale, if less than 20% of responses [35] are the highest or lowest possible response option, then it can be assumed that the scale is capturing the full range of potential responses in the population and that changes over time can be detected [36]. The Kolmogorov–Smirnov test was used to assess normality at the item level. A statistically non-significant result (*p* ≥ 0.05) indicates normality [37]. Construct validity was estimated by correlating item 1: “How would you rate your QOL?” and item 2: “How satisfied are you with your health?” with the four domains in the WHOQOL-BREF using Spearman’s rank correlation.

#### Reliability

Internal consistency was assessed using Cronbach’s alpha. To determine how closely each item correlates with the total score, corrected item-total correlations were calculated. The limit for satisfactory item correlation was set to > 0.30. Values less than 0.30 indicate that the item is measuring something different from the scale as a whole [36].

#### Factor structure

The appropriateness of performing confirmatory factor analysis was checked according to quality criteria. These criteria were fulfilled by means of the Kaiser–Meyer–Olkin measure of sampling adequacy (KMO-MSA) (0.90, should be 0.50 or above), Bartlett’s test (0.01, should be < 0.05) and the determinant of the correlation matrix (0.008, should be > 0.00001) [38]. The number of cases per item was also calculated. Recommendations range from 2 to 20 subjects per item [39, 40], with an absolute minimum of 100 to 250 subjects [41–43]. Confirmatory factor analysis with maximum-likelihood estimation was applied to assess goodness of fit by means of various descriptive fit indices [44]. Specifically, the normed fit index (NFI), the comparative fit index (CFI) and the root mean squared error of approximation (RMSEA) were used [44].

The NFI equals the difference between the chi-square of the two models divided by the chi-square of the null model. An NFI over 0.90 is preferable. An NFI of .90 shows that the model of interest improves the fit by 90% in relation to the null model. CFI assesses fit relative to a null model and ranges from 0 to 1, where values exceeding 0.95 are regarded as acceptable [45], CFI (0.99, 0.95, 0.92 and 0.90) distinguish between excellent, close, fair and mediocre or poor models respectively [46]. The RMSEA test assesses the lack of fit per degree of freedom of the model, “*a cutoff value close to .06 for RMSEA are needed to conclude a relatively good fit between the hypothesized model and the observed data”* [45]*(p.1).* SPSS Amos only accepts data files with no missing values, which meant that 14 participants (*n* = 240) had to be excluded from the confirmatory factor analysis using listwise deletion [47].

## Results

### Validity

#### Internal validity

Overall, the rate of missing data was low and it was less than 2% for the majority of items. Of the 254 participants answered all 25 items. The Kolmogorov–Smirnov test showed a significant result at the item level (*p* < 0.001), which indicates that the data were non-normally distributed. The floor effect ranged from 1 to 5% and the ceiling effect from 11 to 43%; the ceiling effect was above 20% for 21 of 25 items (Table 2).

**Table 2:** Data quality of the WHOQOL-BREF used with next of kin to persons in nursing homes (*N* = 254)

Items	N	Mean (SD)	Median	Missing (%)	Floor (%)	Ceiling (%)
1. Overall assessment of QOL	253	4.2 (.81)	4	0.004	0	38
2. Overall satisfaction with health	253	3.9 (.88)	4	0.004	1	23
3. Pain	254	4.2 (.81)	2	0	2	43
4. Medication	254	3.9 (.88)	2	0	5	43
5. Positive feelings	254	2.0 (1.1)	4	0	1	28
6. Spirituality	254	4.1 (.80)	4	0	1	32
7. Thoughts	253	3.8 (.71)	4	0.004	5	11
8. Safety	254	4.1 (.68)	4	0	2	24
9. Environment	253	4.0 (.63)	4	0.004	3	21
10. Energy	254	3.8 (.89)	4	0	1	42
11. Body image	254	3.9 (.81)	4	0	0	48
12. Finances	252	4.1 (.94)	4	0.008	2	38
13. Information	254	4.3 (.67)	4	0	1	39
14. Leisure	254	3.8 (.97)	4	0	2	42
15. Mobility	254	4.3 (.74)	4	0	2	42
16. Sleep	253	3.8 (1.0)	4	0.004	1	28
17. Activities	253	4.0 (.84)	4	0.004	0	30
18. Work	252	3.9 (.81)	4	0.008	3	25
19. Self-esteem	254	4.1 (.65)	4	0	0	24
20. Relationships	249	4.3 (.66)	4	0.020	0	34
21. Support	251	4.2 (.70)	4	0.010	0	32
22. Home	251	4.5 (.63)	5	0.010	1	56
23. Services	250	4.1 (.80)	4	0.020	1	29
24. Transport	248	4.2 (.88)	4	0.020	2	38
25. Negative feelings	251	2.1 (.76)	2	0.010	1	17

#### Construct validity

Statistically significant positive correlations were found between how the participants rated their overall QOL (item 1) and the four domains in the WHOQOL-BREF instrument (physical health: r_s_ = 0.65, *p* < 0.001; psychological health: r_s_ = 0.67, *p* < 0.001; social relations: r_s_ = 0.44, *p* < 0.001; environment: r_s_ = 0.57, *p* < 0.001). Significant correlations were also found between how the participants rated overall satisfaction with their health (item 2) and the four WHOQOL-BREF domains (physical health: r_s_ = 0.73, *p* < 0.001; psychological health: r_s_ = 0.62, *p* < 0.001; social relations: r_s_ = 0.44, *p* < 0.001; environment: r_s_ = 0.49, *p* < 0.001).

### Reliability

#### Internal consistency

The Cronbach’s alpha of the total instrument was 0.83, indicating satisfactory internal consistency for the overall scale. Table 3 shows the results of the four subscales. The corrected item-total correlations within each scale varied between 0.35 and 0.68 and were thus well above the lower limit of 0.30.

**Table 3:** Internal consistency for each domain in WHOQOL-BREF in relation to previous psychometric testing of WHOQOL-100 and WHOQOL-BREF

Domain	Swedish WHOQOL-BREF (*N* = 254) Cronbach’s alpha	WHOQOL-BREF^a^ Cronbach’s alpha	WHOQOL-100^a^ Cronbach’s alpha
Physical	.86	.80	.86
Psychological	.82	.76	.82
Social relationships	.77^b^	.66	.73
Environment	.80	.85	.85

^a^The WHO-Group (1998)

^b^Only two items, therefore Cronbach’s alpha may not be reliable

### Factor structure

The overall measure of sampling adequacy, using the KMO test, for the WHOQOL-BREF matrix was .90, which is a clear indication that data was appropriate for factor analysis. Additionally, Bartlett’s statistic showed a *p*-value < 0.05 (Bartlett’s statistic = 2916, df = 253, *p* = 0.01), and the number of cases (participants) per item was calculated at 11, in line with the recommendations of 5–10 cases per item [48]. The confirmatory factor analysis showed that the chi-square for the model was significant, indicating an unacceptable model fit. However, according to the CFI, a large amount of the variance was accounted for (CFI = .93), indicating a fair to close model fit. Additionally, an RMSEA value of .06 indicated a good model fit and demonstrated that several significant relations were accounted for (Tables 4 and 5).

**Table 4:** The model statistics of the confirmatory factor analysis of the WHOQOL-BREF when applied to next of kin of older persons in nursing homes (*n* = 240)

Statistics	X^**2**^	df	***P***	CFI	RMSEA
Model fit for WHOQOL-BREF with four dimensions	405.738	207	.001	.926	.063

*X*^2^ Chi-square goodness of fit, *df* degrees of freedom, *CFI* comparative fit index, *RMSEA* root mean square error of approximation

**Table 5:** Factor loadings in confirmatory factor analysis for WHOQOL-BREF

WHOQOL-BREF items	WHOQOL-BREF domains			
	Physical	Psychological	Social relationships	Environment
(3) Pain	0.62			
(10) Energy	0.85			
(16) Sleep	0.47			
(15) Mobility	0.61			
(17) Activity	0.89			
(4) Medication	0.54			
(18) Work	0.83			
(5) Positive feelings		0.70		
(7) Think		0.61		
(19) Esteem		0.70		
(11) Body esteem		0.55		
(25) Negative feelings		0.59		
(6) Spirituality		0.71		
(20) Relationships			0.81	
(21) Support			0.77	
(8) Safety				0.73
(22) Home				0.53
(12) Finances				0.47
(23) Services				0.45
(13) Information				0.51
(14) Leisure				0.75
(9) Environment				0.61
(24) Transport				0.34

## Discussion

The literature shows that our study is the first published paper to investigate the psychometric properties of the Swedish version of WHOQOL-BREF on next of kin to older persons in nursing homes. The results showed that the WHOQOL-BREF dimensionality and factor-loading pattern fits the group and that the measured variables represent the QOL construct in the group. However, a notable ceiling effect may restrict the ability of the WHOQOL-BREF to detect positive changes in QOL over time among next of kin to older persons in nursing homes.

### Validity

#### Internal validity

The quality of the data in this study can be assumed to be satisfactory with regard to the number of participants, *n* = 240, when compared to other psychometric studies performed on similar instruments. For example, in another study using the WHOQOL-BREF among 130 next of kin to patients receiving palliative care [23], the number of respondents can be regarded as the weakness in that study, given the recommendation to have 2–20 participants per item when performing factor analysis [38, 39]. However, many published studies have failed to justify their sample size determination, [48], which highlights the need for clear, scientifically sound recommendations on the topic of optimal study samples when using factor analysis in this kind of study.

The notable ceiling effect of over 20% in 21 of the 25 WHOQOL-BREF items in this study might pose an obstacle when using these items as outcome measures in clinical trials among next of kin to older persons in nursing homes. These items might underestimate actual changes and differences between study participants in this context [49]. The recommended maximum ceiling and floor effect varies in different publications, but a ceiling or floor effect is usually defined as 15% or more [35]. A notable ceiling effect can make changes or differences detectable only in one direction [50]. This characteristic means that most items in the WHOQOL-BREF might only measure negative changes, not positive ones, among next of kin to older persons in nursing homes. One explanation for the high ceiling effect might be the sample homogeneity in age and gender [36]. QOL has, in previous research, shown to vary according to age and sex [51]. In this study, the mean age was 64 years (± 9.7), and 76% of the sample were women, which may have contributed to the high QOL scores. The Swedish National Board of Health and Welfare [51] has demonstrated that it is primarily next of kin aged 30–44 years who are more adversely affected by providing care. In that study, up to 74% of the next of kin experienced the commitment of providing this care as psychologically stressful. Additionally, the results also showed that it is more common for female than male caregivers to experience psychological stress, which contradicts our results [51]. The likely explanation for this discrepancy is that in our study, the women were significantly older and in a different phase of life. Caregivers over the age of 64 and those between 18 and 29 reported stress to a much lower extent. Therefore, if our study had included next of kin in the 30–44 age group, the ceiling effect might not have been so high. Accordingly, there is a need for further research on the psychometric properties of the WHOQOL-BREF among a more mixed group of next of kin to older persons in nursing homes, including those aged 30–44 years.

#### Construct validity

Overall, the result indicates good construct validity of the WHOQOL-BREF. Each domain in the instrument correlated with the participants’ ratings on overall QOL and satisfaction with health, constructs that are theoretically similar. However, these results should be interpreted with some caution because the instrument is based on an ordinal scale without equidistant measurement points, in line with Streiner’s recommendations on the development and use of health measurement scales [36].

### Reliability

#### Internal consistency

The internal consistency for each domain is in line with previous psychometric testing of the WHOQOL-100 and the WHOQOL-BREF (Table 3) and studies previously conducted on the WHOQOL-BREF in a different context [17]. A Cronbach’s alpha of 0.83 indicated satisfactory internal consistency in line with the study by Dalky and colleagues [52], whose results showed satisfactory Cronbach’s alpha (≥0.70) and item-internal consistency (≥0.40). Taken together, based on the results of our study in comparison with previously conducted studies, the variables that comprise the scale can be assumed to measure the same underlying construct. However, when Cronbach’s alpha coefficients were calculated separately for the four WHOQOL-BREF domains, high values for Cronbach’s alpha only showed good internal consistency for three out of four domains. One weakness is that the social relationships domain includes very few items: in the original instrument, it included three items, and in our study, it included only two items after the question “*How satisfied are you with your sex life?*” was excluded. It might therefore be questioned whether the high total Cronbach’s alpha coefficient observed for the entire scale can be interpreted as indicating a unidimensional measure [53]. However, the corrected item-total correlations were well above the limit of 0.30 [36], indicating that the items are measuring the same underlying construct.

### Factor structure

According to the confirmatory factor analysis, a large amount of the variance was accounted for (CFI = .93), indicating a fair to close model fit [44]. Additionally, the RMSEA value of .06 indicated good model fit and that several significant relations were accounted for (Table 4).

The chi-square for the model, however, was significant, indicating an unacceptable model fit. Nonetheless, chi-square goodness of fit has been shown to be sensitive to sample size: the larger the sample size is, the more likely a model will fail to fit when using the chi-square goodness-of-fit measure. Consequently, many researchers disregard this index if the sample size is more than 200 and the other indices indicate that the model is acceptable [54]. In addition, CFI and RMSEA have been shown to be less sensitive to sample size [54]. Based on the result of the factor analysis and in keeping with recommendations for interpreting factor analysis [55], we cautiously interpret the model structure as having a fair to close model fit. Nevertheless, the result of the WHOQOL-BREF factor analysis must be interpreted in the light of the fact that “no confirmatory factor analysis model should be accepted on statistical grounds alone; theory, judgement, and persuasive argument should play a key role in defending the adequacy of any estimated confirmatory factor analysis model” [56] (p. 554).

### Validity and reliability in relation to other settings

In addition to the two intervention studies from Germany and USA, studies on next of kin and have been conducted in Brazil, Italy and Jordan, three countries with a large cultural distance from Sweden [18, 27]. This makes it difficult to compare these results with the results of the present study on next of kin. Firstly, in those countries, the younger generation in the family is traditionally obligated to take care of older family members, whereas in Sweden this care is based on a social welfare system without formal responsibility for the older generation compared with countries where the family is obligated formally. Secondly, no study has included next of kin to older person with multiple morbidities living in a nursing home. Thirdly, the most common psychometric data in these studies are missing or based on a small sample.

### Study strengths and limitations

The study has some strengths and limitations. One strength is that the WHOQOL-BREF is based on a thorough theory and definition, and another is that the participants were recruited from 30 nursing homes, both large and small. However, issues related to our data might limit the findings. The domain of social relationships was not examined completely, as the Swedish ethical review board decided to exclude one of the questions. Another study limitation might be that the CFI value of 0.93 is lower than the recommended value of 0.95 for goodness of fit [46]. However, RMSEA showed good model fit and several significant relations were accounted for and the result of CFI was relatively close to the recommended value. Based on this and in line with the recommendations of Marcoulides and Yuan [46] we conclusively conclude that the model has a fair fit when used among next of kin to older persons in nursing homes. Additionally, the present study’s CFI value of 0.93 is higher than the CFI of 0.90 found in the original psychometric study of WHOQOL-BREF [17]. Another limitation was, as discussed above, the homogeneous sample. Furthermore, confirmatory factor analysis with maximum-likelihood estimation used in this study to enable comparison of the results with previously conducted studies does not take ordinal data into account, which may have affected the results. However, according to Cheng-Hsien Li [57] ignoring the ordinal nature of the data when using the maximum-likelihood estimation may affect the results by yielding underestimation of factor loadings. In turn, this can reduce precision and accuracy of the model, which can lead to misleading conclusions. The fewer participants in the study, the greater the risk of underestimation, but in our study the sample size was relatively large (*N* = 254). Maximum-likelihood estimation is best fitted when the latent distributions are non-normal with a sample size of *N* = 200 [57]. Furthermore, Likert scales, as in WHOQOL-BREF, can be treated as interval data to allow parametric statistics such as correlational analyses, factor analysis and analysis of variance to be used as long as all other design conditions and assumptions are met [58].

## Conclusion

The findings suggest that the WHOQOL-BREF instrument may constitute a reliable and valid measure of QOL for next of kin to older persons in nursing homes. The results of this study imply some uncertainty, since the ability of the instrument to detect changes in QOL over time is constrained by the percentage of respondents responding at the ceiling or floor level of the scale. There is a need to assess whether the WHOQOL-BREF reliably represents and measures QOL in a broader age group of next of kin to older persons in nursing homes.

## Data Availability

The data generated during the project and analysed in this study are not publicly available due to the inclusion of sensitive information regarding a vulnerable group, older persons living in nursing homes. The regional ethical review board in Lund set limitations regarding accessibility of the data. Therefore, before approving access to data, the principal researcher (GA) of the KUPA project must consult with the review board.

## Abbreviations

CFI: Comparative fit index
KMO: Kaiser–Meyer–Olkin
KUPA: *KUnskapsbaserad PAlliativ vård* (knowledge-based palliative care)
QOL: Quality of life
RMSEA: Root mean square error of approximation
WHO: World Health Organization
WHOQOL-100: World Health Organization Quality of Life-100
WHOQOL-BREF: World Health Organization Quality of Life (abbreviated)
